# Ultra-Wideband Sensors for Improved Magnetic Resonance Imaging, Cardiovascular Monitoring and Tumour Diagnostics

**DOI:** 10.3390/s101210778

**Published:** 2010-12-02

**Authors:** Florian Thiel, Olaf Kosch, Frank Seifert

**Affiliations:** Physikalisch-Technische Bundesanstalt (PTB), Abbe-Str. 2-12, 10587 Berlin, Germany; E-Mails: olaf-kosch@ptb.de (O.K.); frank.seifert@ptb.de (F.S.)

**Keywords:** Ultra-wideband (UWB) radar, electrocardiography (ECG), myocardial surface, high and ultra-high field magnetic resonance imaging (MRI), fMRI, multimodal sensing, Finite-difference time-domain method (FDTD)

## Abstract

The specific advantages of ultra-wideband electromagnetic remote sensing (UWB radar) make it a particularly attractive technique for biomedical applications. We partially review our activities in utilizing this novel approach for the benefit of high and ultra-high field magnetic resonance imaging (MRI) and other applications, e.g., for intensive care medicine and biomedical research. We could show that our approach is beneficial for applications like motion tracking for high resolution brain imaging due to the non-contact acquisition of involuntary head motions with high spatial resolution, navigation for cardiac MRI due to our interpretation of the detected physiological mechanical contraction of the heart muscle and for MR safety, since we have investigated the influence of high static magnetic fields on myocardial mechanics. From our findings we could conclude, that UWB radar can serve as a navigator technique for high and ultra-high field magnetic resonance imaging and can be beneficial preserving the high resolution capability of this imaging modality. Furthermore it can potentially be used to support standard ECG analysis by complementary information where sole ECG analysis fails. Further analytical investigations have proven the feasibility of this method for intracranial displacements detection and the rendition of a tumour’s contrast agent based perfusion dynamic. Beside these analytical approaches we have carried out FDTD simulations of a complex arrangement mimicking the illumination of a human torso model incorporating the geometry of the antennas applied.

## 1. Introduction

If an electromagnetic wave interferes with the human body, it propagates through it and is reflected at interfaces between tissue materials with different dielectric properties. Therefore, biomedical applications of ultra-wideband (UWB) radar, which comprises a spectral bandwidth up to 10 GHz with *P*_rms_ ∼ 4 mW in this frequency band, promise a very important means to remotely monitor physiological signatures like myocardial deformation and respiration.

The sensitivity of these sensors to ultra-low power signals makes them suitable for medical applications including mobile and continuous non-contact supervision of vital functions. The latter one is an essential point, since each critical care unit is equipped with a multitude of devices necessary to monitor the state of the patient. Since non-ionizing radiation is used, and due to the ultra-low specific absorption rate (SAR) applied, UWB techniques permit noninvasive sensing at no risk, in contrast to catheter or X-ray techniques. Non-contact detection and monitoring of human cardiopulmonary activity through bedding and clothing would be a valuable tool in sleep monitoring, home health care applications and for the combination with other modalities already established in clinical medicine, to gain complementary information. Most alternatives to standard heart and respiration monitors need leads and contacts and often require accurate control or placement. A vital signs monitor that can sense contactlessly and through hair and clothing would be ideal in these situations [1].

The acquisition of vital functions applying microwaves is obvious and was shown by many authors, e.g., [1–7], to name but a few. These investigations mostly focus on the presentation of the physiological time courses reconstructed from the applied microwave method without providing a physiological interpretation of the signal’s shape [1,2] and [3]. Several attempts have been made in the past to show the interrelation of reflected microwave signals from the human thorax and simultaneously acquired ECG data, e.g., [4] and [5]. These studies restricted their investigation on analysis of overall correlation between the two modalities or focuses on the detection of heart rate variability by the applied radar method focusing on surface displacement. Other contributions avoid the problem of microwave signal damping and dispersion while propagating through the different tissues of the thorax by an un-physiological *ex-vivo* investigation of excised hearts [6]. Unfortunately non of those carried out an in depth interpretation of the correspondence between the different ECG episodes and the mechanical data, which is the prerequisite for understanding and in our case the basis to identify landmarks for triggering a magnetic resonance scanner. Even one of the latest publications related to this topic [7] concludes that the community is still in need of further interpretations of the electro-mechanical coupling presented by an ECG/microwave approach.

Our approach to combine ultra-wideband sensors and magnetic resonance tomography for the benefit of biomedical research and clinical medicine is novel, and the advantages it offers, beside further suggested stand-alone applications of the broadband sensors, will presented within this review article.

Magnetic resonance imaging (MRI) is the most important tool in modern cardiology and neuroscience. Due to the continuing improvements of the spatial and temporal resolution of this imaging modality, great progress could be made in the field of brain science and the imaging of cardiovascular diseases. Nowadays, however, a point has been reached where further improvements in resolution are limited. The signal to noise ratio in MRI increases approximately linearly with increasing static magnetic field, therefore the use of so-called high (B_0_ ≥ 3 T) and ultra-high field (B_0_ ≥ 7 T) systems can overcome these limits and facilitate new findings about the human brain and the heart. Our research aims at the implementation of ultra-wideband sensors for biomedical applications. To this end we seek to exploit the synergetic use of UWB remote sensing combined with MRI, to gain complementary information, e.g., to accelerate and improve cardiac MRI.

The application of UWB systems together with a MRT is not a simple task, but requires compatibility considerations [8,9]. The ambient conditions inside a MR scanner are defined by three different types of fields. First, a static magnetic field of *B*_stat_ = 1.5 − 7 T, generated by a superconducting coil, provides a reference orientation of the nuclear spins of the regions under inspection. Gradient magnetic fields with a slope of d*B*_grad_/d*t* = 50 T/s at the rising edge are switched during diagnostic measurements, to provide the required tomographic molecular spectra. Furthermore MRI is based on the resonant excitation of protons, which implies a very narrow excitation bandwidth (125 MHz ± several kHz at 3 T) with fields in the kW range. On the other hand an UWB device excites a material under test with signals offering a bandwidth of several GHz, but the applied integral power lies below *P*_rms_ ∼ 4 mW in this particular frequency band. The SNR of a MR scan is not affected by the UWB signals, since the receiver bandwidth of 10 kHz to 100 kHz is very low compared to the GHz bandwidth of the UWB system, moreover the antennas attenuate the transmitted UWB signal at 125 MHz, the Larmor frequency of protons at 3 tesla, by more than 100 dB. Comparing MR images taken from a MR head phantom with and without UWB exposure, within measuring uncertainty, no additional noise could be observed. So, according to expectation, the MRI system was not affected by the UWB signals, as these appear as a low power noise source to the MR system. Nonetheless special precautions must be taken to reduce eddy currents in the UWB antennas. The gradient fields induce eddy currents in the metallised sections of the antenna according to the Faraday’s law of induction. In turn, these eddy currents interact with the static magnetic field by exerting a mechanical torque on the antenna structure. We have solved these problems in sufficient detail in [8] and [9], so we will focus on the biomedical applications in this article.

Physiological noise, like respiratory and cardiac displacements, introduces motion artefacts in the MR image. We have already established a combined MRI/UWB prototype demonstrating the absence of any mutual interference between both systems, proving the feasibility of the UWB radar method to monitor respiratory and myocardial displacements in a 3 T scanner [9]. Additionally, we have validated the physiological signatures monitored by UWB-radar, utilizing reference signals provided by simultaneous MR measurements on the same subject [10]. Especially the ability to monitor non-invasively the motion of organs within the human body was shown. With an MR-compatible UWB radar, the characteristic landmarks of the heart muscle, the thorax or the brain/skull during breathing could be followed without disturbing the actual MR measurement. In the following, we offer a partial review of our activities, exploring the benefits of UWB radar for high- and ultra-high field MRI. In all experiments, described below, we applied an M-sequence UWB radar system (spectral bandwidth of about 5 GHz) which transmits a periodic pseudorandom waveform [11]. Furthermore theoretical investigations were carried out, utilizing an analytical model of the electromagnetic wave propagation in dispersive, stratified biological objects, to prove the feasibility of this ultra-wideband approach to biomedical challenges like intracranial oscillation detection to improve high resolution brain imaging, and to confirm the benefit for the detection of, e.g., intracranial tumours by its perfusion dynamic. Finally, Finite-difference Time-Domain method (FDTD) simulations will help us to improve our understanding of the electromagnetic field distribution inside and outside the human thorax in different episodes, aiming at the further improvement of our signal processing algorithms.

## 2. Ultra-Wideband Radar

**Figure 1.**
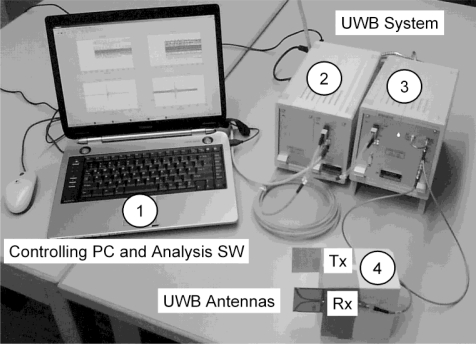
Prototype of our MR-compatible UWB system (MEODAT, Ilmenau), designed according to the Medical Device Directive, in a basic set-up for ultra-wideband measurements. **(1)**: Controlling PC and analysis software. (**2)**: Galvanic isolation. **(3)**: M-Sequence Baseband Module (DC-5 GHz). (**4)**: Tx/Rx: transmitting/receiving antenna

The applied prototype of our MR-compatible UWB system (MEODAT GmbH, Ilmenau, Germany), designed according to the Medical Device Directive, comprising galvanic isolation, M-Sequence baseband module (DC-5 GHz), controlling PC with analysis software, and UWB antennas in a bi-static arrangement are depicted in Figure 1.

**Figure 2.**
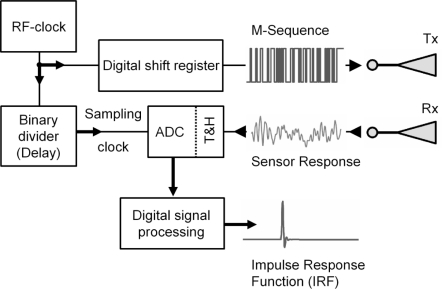
Basic structure of the measurement head in block schematics based on the maximum length binary sequence (M-sequence) [12,13]. T&H: Track and hold circuit. Tx, Rx: transmit and receive antenna.

The goal of UWB radar is to obtain the impulse response function (IRF) of a certain object under test (Figure 2). The UWB controller provides cross-correlation data **R_xy_**(τ) from the transmitted and received signals. The UWB signals cover a very broad frequency range, and can therefore be approximately described as white noise. Therefore, if a linear, time-invariant system, with impulse response function (IRF) *h*(τ), is excited by a stochastic process with autocorrelation φ*_xx_*(τ) ≈ δ(τ), *i.e.*, Dirac’s delta, it follows for the cross-correlation R_xy_(τ) ∼ *h*(τ).

The quality of a measured impulse response function *h*(τ) is mainly determined by the ability to separate closely located peaks and to avoid the masking of smaller peaks due to noise or saturation effects caused by larger signals. The classical UWB approach is based on impulse excitation, which implies that the whole transmission chain is subjected to high peak power. In order to stress the electronics evenly, it is preferable to use continuous wideband signals due to the reduced crest factor. Typical examples of such signals are swept or stepped sine waves, random noise [14], or pseudo-noise (PN) sequences. However, this kind of target stimulation will not provide the IRF directly. It rather requires an appropriate impulse compression technique (*i.e.*, Fourier transform, correlation, or matched filtering), which is often the challenge for the different system concepts. After impulse compression, e.g., correlation in our case, the spectral energy distribution of the correlation result reflects the frequency characteristic of the object’s IRF. Thus, the best one can do is to carry out impulse compression in the digital domain. The digital dynamic range is only limited by the utilized data format which can usually be selected freely. An UWB concept dealing with continuous wave excitation, a largely reduced analog circuit part, and a minimum of components was first introduced in 1999 [15]. It provides maximum length binary sequence (M-sequence) signals to stimulate the test objects, which optimize the crest factor and therefore solve the overload and saturation problem. This original approach forms the basis for different extensions and improvements. The basic structure of such an ultra-wideband radar module is described below.

### 2.1. M-sequence instead of broadband pulses

The basic idea of a maximum length binary sequence (M-sequence) device initially intended for baseband operations at, e.g., 0 to 5 GHz is known from a couple of former publications (see [12,13]) and the theory of pseudorandom codes and their application is given in, e.g., [16] in an introductory form. The basic structure of a wideband M-sequence device is presented in Figure 2. The M-sequence—the stimulus signal for the object to be investigated—is generated by a digital shift register, which is addressed by a stable RF clock with frequency *f*_c_. The capturing of the measurement signal is accomplished by using a sub-sampling approach. One of the most important features of the M-sequence approach is that the actual sampling rate *f*_s_ can be derived in a simple and stable way (*i.e.*, by a binary divider) from the RF master clock such that *f*_c_ = 2*^n^f*_s_ (see Figure 2 and Reference [13] for details).

Time-domain measurements use correlation processing in order to gain the wanted impulse response function of the material under test (MUT). In that case, unwanted external narrow band perturbations will be spread over time, since they are not correlated with the test signal. In this way, they cause the same effect as white noise, which is often less critical than a corruption of the whole waveform in time domain, in the case of a classical time-domain reflectrometry (TDR) analysis, or the strong perturbation of individual frequency bands, in the case of network analysis. The following comprises the application of the described system.

## 3. Tracking of Involuntary Head Motions for High Resolution MRI

Subject motion appears to be a limiting factor in numerous MR imaging applications especially at high and ultra-high fields, e.g., high-resolution functional MRI (fMRI), which measures the haemodynamic response related to neural activity in the brain or spinal cord, or Diffusion Tensor Imaging (DTI), that enables the measurement of the restricted diffusion of water in tissue in order to produce neural tract images instead of using this data solely for the purpose of assigning contrast or colours to pixels in a cross sectional image. For head imaging, the subject’s ability to maintain the same head position for a considerable period of time places restrictions on the total acquisition time. This period typically does not exceed several minutes and may be considerably reduced in case of pathologies. In particular, head tremor, which often accompanies stroke, may render certain high-resolution techniques inapplicable. Several navigator techniques have been proposed to circumvent the subject motion problem.

**Figure 3.**
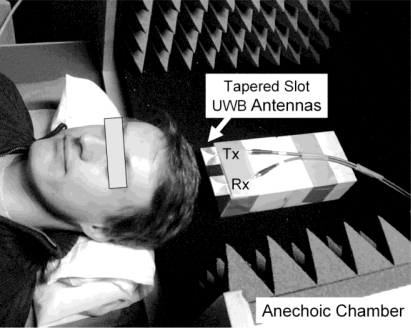
Spontaneously breathing volunteer in supine position, exposed to UWB signals in the GHz range using transmitting (Tx) and receiving (Rx) tapered slot antennas (Vivaldi type).

Optical techniques suffer from resolution issues, cannot penetrate dense body hair (head) and are incapable of detecting the motion of inner organ. MR navigators, however, not only lengthen the scan because of the time required for acquisition of the position information, but also require additional excitation pulses affecting the steady state magnetization. Furthermore, if the very high spatial resolution offered by ultra-high-field MR scanners shall be exploited, the displacements caused by respiration and cardiac activity have to be considered. Thus, we propose applying an UWB radar technique to monitor involuntary head displacements. To monitor head motion induced by respiration and cardiac contraction, we positioned a volunteer in supine position at the opening of an anechoic box (Figure 3).

**Figure 4.**
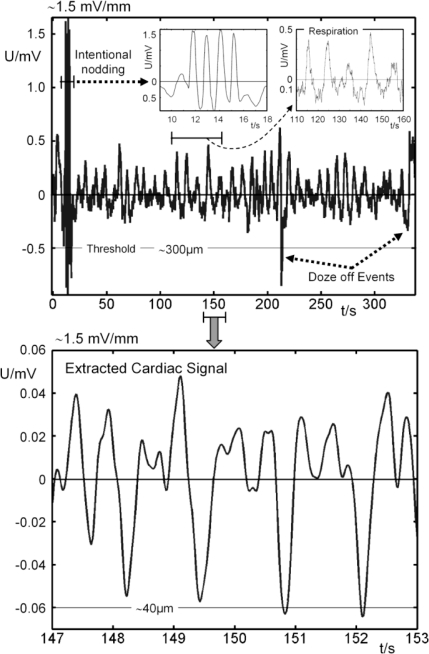
*Top:* motion reconstructed from a measured time interval of 350 s. The left inset displays the four nodding events (∼1 mm amplitude, episode [t = 10 s,…,t = 18 s]) to localize the surface of the head. Respiratory displacements are clearly visible (right inset, episode [t = 110 s,…, t = 160 s]) and spontaneous twitches are highlighted. *Bottom:* selected filtered time interval from the upper time course, showing the cardiac induced displacements.

The MR-compatible tapered slot UWB-antennas (Tx/Rx, Vivaldi type) [17] are positioned perpendicular to the vertex where larger vessels rarely occur. The amplitude of the intentional noddings were measured by the horizontal change in distance between the antennas and the head’s surface prior to the UWB measurement. In this way, the pulsation signals from subcutaneous vessels, especially from those of the side of the head, the throat, and the brain stem can be excluded. In contrast to optical techniques, the volunteer’s hair does not affect the measurements. The resulting cross-correlation data **R_xy_**(τ) from the transmitted and received signals provide information of the propagation time τ necessary for the electromagnetic pulse to reach the air/skin-interface of the vortex. First *in-vivo* motions reconstructed from a measured time interval of 350 s are shown in Figure 4. Since the UWB signals are very sensitive to interface displacements (< 0.1 mm), we used four small nodding events of about 1 mm in amplitude to localize the position of the head in **R_xy_**(τ). The displacement of this reference point was then analyzed by observation of the (relative) variation of **R_xy_**(τ) over time. Thus we could detect all kinds of involuntary motions (respiratory, cardiac), even doze-off-events are visible, demonstrating the feasibility of interfacing a MR scanner with an external UWB radar based motion tracking system. Our system is capable of determining the position of interest with sub-millimeter accuracy and an update rate of 44 Hz. Using the UWB tracking data of the volunteer’s head, the motion artefacts can be compensated in real time or by post-processing, enhancing the actual resolution of the MR scan.

### 3.1. Intracranial pulsation detected

It is well known that simultaneously to the head’s oscillations intracranial oscillations with spatial varying amplitude also occurs, induced by the same physiological sources mentioned above [18].

**Figure 5.**
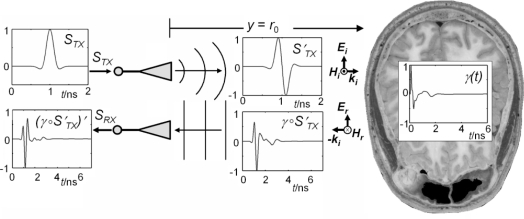
Sketch of the signal path model for the current transfer function *S*_Rx_/*S*_Tx_. *S*_Tx_: excitation signal. *S*′_Tx_ = *E*_i_: free space signal in the channel. Planar linearly polarized electromagnetic wave: *E*_i_/*E*_r_, *H*_i_/*H*_r_: incident/reflected electrical/magnetical field component. *k_i_*: wave vector of the incident wave. γ: impulse response function (IRF) of the multi-layered dielectric structure. γ ○ *S*′_Tx_ = *E*_r_: reflected electrical field component. *S*_Rx_ = (γ ○ *S*′_Tx_)′: received current signal/pulse. There ○ represents the convolution operator. Anatomic slice of the human head taken from [19].

**Figure 6.**
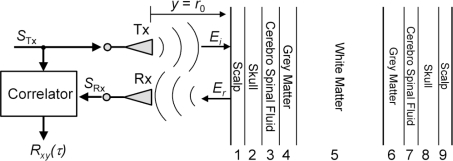
UWB radar probing a multilayered dielectric structure (bi-static set-up). *S*_Tx_/*S*_Rx_: transmitted/received signal; Tx/Rx: transmit/receive antenna; *E*_i_/*E*_r_: incident/reflected electrical field component; *R*_xy_(τ): correlation result from UWB device.

Hence, it is only consequent to ask whether these oscillations are detectable by UWB radar. Due to the simultaneous occurrence of the intracranial displacement and the vibration of the whole head, decomposing both signals requires sophisticated methods. As an initial step towards the solution of this challenge, we need to get a feeling of the change which is introduced in the acquired reflection signal by an intracranial oscillation when exposing the human head to ultra-wideband electromagnetic signals. To this end, we applied an analytical approach [20], which models the signal path and the oscillating stratified arrangement of the brain to get signals free of any interfering compositions. Figure 5 and, in a more abstract way, Figure 6, depict the set-up commonly used to probe the human body with a UWB device.

The antennas are co-polarized and the normal incidence of the EM-wave is assumed. The body can be assumed to form a multilayered dielectric structure with a characteristic reflection coefficient Γ(ω). The UWB signal, which can be a pulse or a pseudo-noise sequence of up to 10 GHz bandwidth, is transmitted utilizing appropriate pulse-radiating antennas Tx (e.g., horn or tapered slot antennas). The reflected signal is detected by Rx and calculating the correlation between received signal *S*_Rx_ and transmitted signal pulse *S*_Tx_ is usually the first step in further signal-processing.

We model the human head from nine planar isotropic dielectric layers, those arrangement as well as individual thicknesses approximate a trans-cranial slice from the Visual Human data set [19] (Figure 5, and Figure 6).

The spectral response of a dielectric medium is appropriately described in terms of multiple Cole-Cole dispersion which, with a choice of parameters appropriate to each constituent, can be used to predict the dielectric behavior over the desired frequency range [21]. For such a layered arrangement the reflection response Γ(ω) can be recursively calculated using an iterative formulation published by [22]. In this way the response of Γ(ω*, t*) to the variation of a certain internal interface can be analyzed. If the stratified object is located at a distance *r_0_* from the Tx/Rx-antennas, which are assumed to be positioned in their mutual far-field, the ratio of the E-fields in the frequency domain at Rx and Tx becomes, assuming a TEM-wave [20]:

(1) $$\frac{E_{Rx}}{E_{Tx}}(\omega )=\Gamma (\omega )\cdot \left[a(r_{0})\cdot e^{\left(-2j\omega \frac{r}{c_{0}}\right)}\right]∼\frac{S_{Rx}(\omega )}{{\left(j\omega \right)}^{2}S_{Tx}(\omega )}$$

The two folded time integration considers the ideal transfer function of the transmitting and receiving antenna, respectively (ideal case).To account for the path dependent damping of the electromagnetic wave in the far-field *a*(*r_0_*) is introduced. Assuming a spherical wave reflected at a plane, extended surface, *a*(*r_0_*) = (2*r_0_*)^−1^.

A typical class of broadband electromagnetic excitation pulses are those of the Gaussian shape and their derivatives [23]. We used a modified Ricker pulse, which is the second derivate of the Gaussian pulse. A broadband pulse, whose spectral behavior is similar to the excitation signal we apply in our envisaged medical application (flat spectrum down to several MHz, −3dB at 10 GHz), can be formed from the so-called Ricker pulse (Figure 5, *S*_Tx_). Theoretically the received signal *S*_Rx_ in the frequency domain becomes [20]:

(2) $$S_{Rx}(\omega )=\left[S_{Tx}\cdot H_{Tx}\cdot H_{Rx}\cdot \Gamma (\omega ,\;t)\cdot a(r_{0})\cdot e^{\left(-2j\omega \frac{r_{0}}{c_{0}}\right)}\right]$$

where *H*_Tx_ and *H*_Rx_ denotes the transfer function of the transmitting and receiving antenna, respectively.

**Figure 7.**
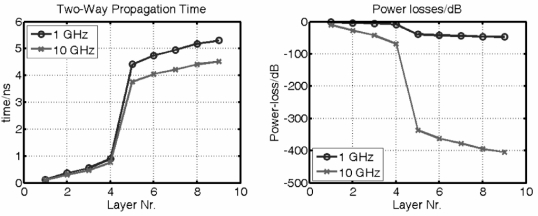
Two-way propagation time and one-way power losses of the layered human head model: *Left:* calculated propagation time after each layer for two different frequencies. Propagation times to reach a certain interface are indicated by the data points. The lines are guides to the eye. *Right:* power losses after each layer of the electromagnetic wave while traversing the 9-layer model computed for two different frequencies.

From the layered model we can calculate the evolution of the propagation time and power losses while the electromagnetic wave traverse each layer at different frequencies (see Figure 7). The two-way propagation time to reach layer 5, *i.e.*, the white matter, is τ_5_ ∼ 0.7 ns (see Figure 7, left).

In the following we will simulate the physiological event by variations of Γ(ω, *t*), which is done by a sinusoidal oscillation of the white matter (layer 5) by an amplitude of 1 mm. Accordingly, the Cerebro Spinal Fluid (CSF, layer 3 and layer 7) varies antipodal.

**Figure 8.**
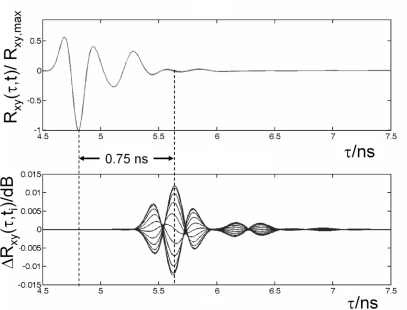
*Upper*: normalized overlapped correlation results *R*_xy_(τ, *t*)/ *R*_xy_,max gained from a sinusoidal oscillation of the intracranial white matter (10 discrete time steps per period). *Lower*: overlapped variation Δ*R*_xy_(τ*, t_i_*) in *R*_xy_(τ) for each elongation, *i.e*., each time step *t*_i_. The envelope of Δ*R*_xy_ corresponds to a 1 mm elongation. Indicated by Δ*t* = 0.75 ns is the two-way propagation time below the surface of the model where the maximum is found.

The calculated correlation result *R*_xy_(τ, *t*) gained from the simulation and the variation Δ*R*_xy_(τ*, t_i_*) after a certain propagation time are depicted in Figure 8. For a maximum intracranial amplitude of 1 mm the variation becomes Δ*R*_xy_(τ*, t_i_*)∼0.01 dB at a propagation time where the white matter is located (τ_5*_∼0.7 ns, see Figure 8). Hence, requesting a high-fidelity receiver. The reconstruction of the intracranial motion applying the reconstruction algorithm proposed in [20] gave us a maximum deviation from the reference oscillation of about 4 %. We conclude that the detection of intracranial oscillations using non-contact UWB is indeed feasible [24].

It must be noted that for all real medical applications of this broadband technique trying to monitor variations of the bodies interior, sophisticated signal processing techniques must be applied to decompose signal originating from the body’s surface and signals originating from deeper sources. Such techniques are multivariate analysis techniques such as Principal Component Analysis or, demanding much stricter constrains, Independent Component Analysis. Both techniques were successively applied by our group [10,25]. Another issue for real applications is the influence of the antennas transfer function, which was assumed to be ideal in the above simulation. If the influence of the antennas is extracted from the received signal by using proper de-convolution techniques, which also can be derived and optimized utilizing our model, the time courses of the ideal channel can be regained.

## 4. Navigator for Cardiac MRI

MRI often uses electrocardiography (ECG) information to acquire an image over multiple cardiac cycles by collecting segments of *k*-space at the same delay within the cycle. This requires breath hold as it is assumed that cardiac positions are reproducible over several ECG cycles, but is not, as we will see in the following from our findings. Unfortunately, in clinical situations many subjects are unable to hold their breath. High resolution MRI acquisition in the free-breathing state is of high clinical relevance, as haemodynamic parameters may differ between breath holding and free breathing. At high and ultra-high magnetic fields, ECG triggering is additionally hampered by the corruption of the ECG due to the magnetohydrodynamic effect (MHD) [26]. The MHD effect generates voltages, mainly related to aortic blood flow in a strong static magnetic field, which are superimposed on the ECG used. Executing MRI in a pre-defined state of the heart cycle, e.g., end-systolic or end-diastolic, the definition of landmarks in the acquired UWB signatures, which offers a mechanical representation of the electrically activated myocardium, is demanded for controlled MRI data acquisition. For this purpose, a stand-alone analysis of physiological signals acquired by UWB radar is essential, and the cardiac motion signals must be interpreted by comparison with established clinical reference techniques (e.g., high-resolution ECG).

**Figure 9.**
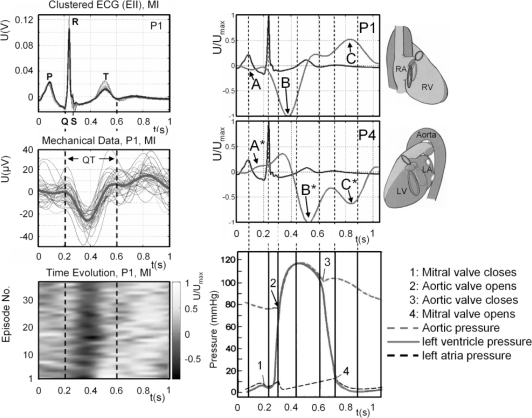
*Left:* Evolution of the reconstructed surface displacement intervals measured in a breath hold (MI) in the selected positions. *Right:* Comparison of the normalized time courses reconstructed from two exposure positions (P1, P4) measured during maximal inspiration and corresponding ECG. Wiggers diagram: 1: Mitral valve closes (stops filling of left ventricle), 2: Aortic valve opens (left ventricular volume is ejected), 3: Aortic valve closes, 4: Mitral valve opens (filling of left ventricle). A/A*: contraction of right/left atrium. B/B*: contraction of right/left ventricle. C/C*: filling of right/left ventricle. Anatomical sketch and pressure curve taken and adapted from [28].

The interrelation between the electrical and mechanical activities of the human heart is known to be strong and is very well investigated using the ECG together with other modalities, e.g., ultrasound, or invasive, catheter based methods. Time intervals in the ECG are closely related to the mechanical activity of different parts of the heart. This part of the article deals with the variations of the signals reconstructed from UWB radar measurements if the myocardial muscle is illuminated from different well-selected spatial directions [27] and the ECG is simultaneously measured. These positions are the so-called radiographic standard positions, which are illumination positions of the heart in an appropriate transversal plane (height of the 8th segment of the thoracic spine, Th8), at the right-hand side, from an angle of 45° (P1, mainly the right ventricle is illuminated) towards 180° (P4: left side view, mainly the left ventricle is illuminated), respectively (see, Figure 9, right time courses and anatomical sketch of the heart). Application of UWB radar from these positions illuminate different parts of the myocardial surface, corresponding to different functional units, and, therefore, should deliverer different contraction patterns. We have clustered the simultaneously acquired ECG of each measurement (∼40 s) and calculated the median. The same processing was applied to the corresponding reconstructed UWB time courses (Figure 9, left). The overlapped ECG intervals and its median value in Figure 9, left (bold grey line) and the corresponding overlapped epochs form the reconstructed mechanical time course and its median value (bold grey line) is juxtapositioned for the illumination positions P1 (right ventricle) and P4 (left ventricle). Additionally the juxtaposition of the time evolution of the mechanical epochs for each position is added to give more information about the variation of the mechanical data. The lower right time course of Figure 9 is a part of a Wiggers diagram comprising the pressure in the left ventricle (bold line), the aortic pressure (dashed bold line), and the pressure in the left atria (thin dashed line).

Comparing the time courses, we observed that the global variance of the clustered, extracted signals varies remarkably between the electrical activation and the mechanical response, displayed by UWB radar. This is in good agreement with the experiences from clinical ultrasound investigations of cardiac contractility variability, and indicates that the myocardial activity is not only controlled by the electrical excitation, but also by other metabolic factors (e.g., hormonal, oxygen level, temperature regulation, *etc.*).

To evaluate the surface UWB time courses, a standard Wiggers diagram (WD) [28], comprising the pressure in the different cardiac functional units in marked contraction phases, is juxtaposed to the recorded ECG epochs (Figure 9, lower right). As expected, the mechanical representation varies depending on the illuminated superficial part of the myocardium. Nevertheless, similarities can be identified. Most eye-catching is the strong change of signals in the course of the systolic phase (start at 1, end at 3 in the WD, QT interval in the ECG, Figure 9). Due to the contraction of the myocardium (∼−25% of diameter), the radar cross-section decreases accordingly. Additionally, the contraction pattern of different functional units can be distinguished by examination the different illumination directions. For instance, the contraction of the right/left atrium (see Figure 9, A/A^*^), the contraction of the right/left ventricle (Figure 9, B/B^*^) and the filling of the right/left ventricle (Figure 9, C/C^*^) can be distinguished from the two positions P1 (right anterior oblique, RAO) and P4 (left lateral, LL). The coarse physiological temporal evolution of the cardiac cycle is as follows: (i) contraction of the right atrium, (ii) contraction of the left atrium, (iii) contraction of the right ventricle, (iv) contraction of the left ventricle, (v) filling of the ventricle. Therefore, the delayed contraction of the left functional units with respect to the right one is rendered correctly (Figure 9, A/A^*^, B/B^*^, C/C^*^). This retardation is physiological, for each mechanical activity is preceded by the electrical polarization/depolarization of the myocardium. In standard clinical 1.5 T MRI systems, ECG triggering can be sufficiently well applied for imaging the heart, for the variability of cardiac position (physiological noise) over several cycles can be regarded to be below the resolution of the MRI system (not regarding the measurement duration). With high and ultra- high field systems entering clinical praxis and research, other motion tracking techniques are required, since the resolution of theses systems can only be exploited if contraction cycles with appropriately smaller variance are chosen, especially in a free-breathing scenario. We have investigated whether UWB radar provides the mechanical data from the myocardium and holds the potential to serve as such a navigator technique. We have shown that the mechanical representation of the cardiac contraction provided by UWB radar varies depending on the cardiac contour and the illuminated superficial part of the myocardium (according to Figure 9, right). The results are very satisfactory and prove the ability of UWB radar to monitor physiological events directly at their origin inside the body. From further investigations we could propose a method utilizing blind source separation, which enables us to reliable determine trigger points in the cardiac UWB signal, which are in a fixed delay to the corresponding R-peak of the simultaneous measured ECG. Even the detection of spontaneous changes in the cardiac mechanics seems to be possible (detection of extrasystoles) [25]. We can conclude that UWB radar is capable of triggering the MR data acquisition in several phases.

## 5. High Static Magnetic Fields an their Influence on Myocardial Mechanics

As mentioned above, ECG is extensively used for triggering MR data acquisition to image the heart in a certain stage of contraction or to prevent reduction of image quality by motion artefacts generated by the strong non-linear motion of the heart muscle. Unfortunately, there is increasing difficulty to use the ECG for MR-triggering especially at B_0_-fields beyond 1.5 T (MHD) [26].

If the conducting particles of the blood are redirected by the magnetic field, it is only consequent to ask whether the electrical excitation spread over the myocardial muscle, which is based on ionic transportation, is redirected, too. Such a redirection should be visible in the ECG and in a deviant myocardial contraction, which is directly dependent on the spatio-temporal devolution of the excitation spread. Unfortunately, investigating this effect using the ECG itself is not possible since it is dominated by the MHD effect. Thus, we propose to use UWB radar to monitor the global myocardial dynamics inside an MR scanner. We simultaneously acquired ECG and UWB radar data a) at *B*_0_ ≈ 0 T (earth’s magnetic field), b) at *B_0_* = 1 T at the edge of the bore of a 3-T MR scanner, and c) at *B_0_* = 3 T in the iso-centre of the scanner. A volunteer was positioned in supine position and was asked to hold his breath to exclude breathing artefacts. The MR-compatible, tapered slot UWB-antennas (Tx/Rx) [17] were positioned about 150 mm above the sternum in an appropriate plane through the heart (height of the 8^th^ segment of the thoracic spine). The position of ECG electrodes and the position of the UWB antennas were not changed between the three different measurements. The resulting reflected UWB signal is a superposition of multiple reflections. In the simple case of well separated tissue interfaces, cross-correlation data **R_xy_**(τ) from the transmitted and received signals provide information of the propagation time τ necessary for the electromagnetic pulse to reach each interface [11].

**Figure 10.**
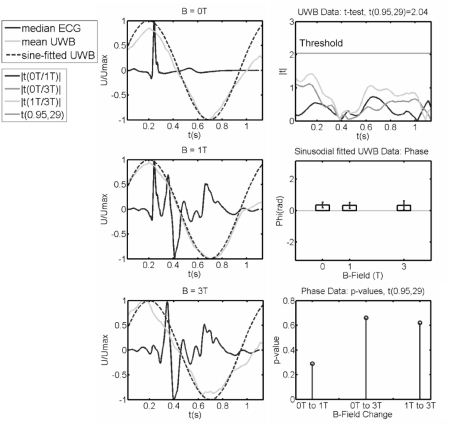
*Left:* comparison of the median of 30 ECGs (EIII), the corresponding reconstructed mean UWB signals and the mean of the sinusoidal fitted UWB data for *B_0_* = 0T, 1 T and 3 T. *Upper right:* result of the *t*-test for each time step in *t* = [0,…,1.1 s]. *Middle and lower right*: comparison of phase of the fit and p-values of the *t*-test on the phase.

Interfaces with high dielectric contrast, e.g., fat/muscle, as they occur at the body surface and the surface of the heart, dominate **R_xy_**(τ). Since we are interested in the movements of selected interfaces we observe the variation of **R_xy_**(τ) over time for each τ by a covariance analysis (see [9,10]). Figure 10 depicts the median of n = 30 ECGs, the corresponding reconstructed UWB signals representing the global cardiac dynamics and the mean sinusoidal fit of the UWB data for *B_0_* = 0T, 1T, and 3T, respectively.

To investigate, whether there is a significant change in the global myocardial mechanics between zero field and 1 T or 3 T we applied Student’s *t*-test [*t*(0.95,29), paired, two-sided] on the UWB data for each time step in the standardized ECG epoch *t*=[0,…,1.1 s]. We found no statistically significant change between all three UWB measurements. Additionally, the phase of the sinusoidal fit was compared applying a *t*-test [*t*(0.95,29)] to check for significant excitation delays. Again, there was no significant change in phase for increasing field. We found *p*-values well above the significance level (see Figure 10, right panel), represented by α = 1-erf(n/√2) = 5% ( *p*(0T/1T) ≈ 0.29, *p*(0T/3T) ≈ 0.66, *p*(1T/3T) ≈ 0.62). Thus, we cannot reject the null hypothesis, which states there is no significant change between measurements.

## 6. Perfusion Dynamics for Tumor Detection

The dielectric constant of, e.g., malignant breast-cancer, was found to be enhanced by a factor of five compared to benign breast tissue, in the frequency range 0.1…10 GHz [29–32]. Such a contrast provides a solid basis for the application of UWB techniques to medical tomography. Tumour detection is based on the exploitation of the dielectric contrast between tumour tissue and the tissue in it’s vicinity [32,34]. This approach requires absolute permittivity values (Re{ε(ω) = ε′(ω)-i·ε″ (ω)}) whose accurate measurement is hampered by several factors including skin reflection, object’s geometry and depth of the tumour. UWB radar is extremely sensitive to the slightest change in the object’s reflection coefficient Γ(ω*, t*).

This sensitivity is more pronounced towards the body surface and, due to dispersive effects, decreases with increasing depth of the source within the body. Approximating the human body as a stratified object, Γ varies according to the temporal evolution of layer thickness and the change in dielectric properties, both induced by physiological or patho-physiological events [35]. For the detection of tumours, the application of a contrast agent to visualize the perfusion dynamic is common praxis in numerous imaging modalities, e.g., MRI or biomedical optics. In physiology, perfusion is the process of nutritive delivery of arterial blood to a capillary bed in the biological tissue. Perfusion dynamics of a contrast agent reefers to the process of enrichment and subsequent wash-out of this tracer substance, e.g., in a tumour, which was conveyed or dragged with the delivered blood to this particular sort of tissue. Due to the high perfusion of several types of tumours, its contrast agent concentration exceeds the concentration in neighbouring tissue. Thus, the dielectric properties of the contrast agent will influence the tumour’s dielectric properties and therefore the object’s reflection coefficient Γ(ω*,* ε′). Furthermore, the perfusion dynamic (enrichment and subsequent wash out) lies in the time range of minutes. All this encourages us to pursue the detection of tumours by UWB radar, utilizing the change in dielectric properties of the tumour over time by injection of an appropriate contrast agent into the vascular system.

**Figure 11.**
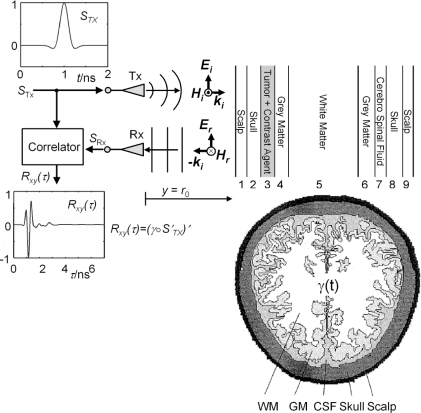
The human head probed by a bi-static set-up. Intracranial tumour in layer 3. WM/GM: white/grey matter, CSF: cerebro-spinal fluid, *T*x/*R*x: transmit/receive antenna; *S*_Tx_: excitation signal. *S*′_Tx_ = *E*_i_: free space signal in the channel. Planar linearly polarized electromagnetic wave: *E*_i_/*E*_r_, *H*_i_/*H*_r_: incident/reflected electric and magnetic field component, respectively. *k_i_*: wave vector of the incident wave. γ: impulse response function (IRF) of the multi-layered dielectric structure. γ ○ *S*′_Tx_ = *E*_r_: reflected electrical field component. *S*_Rx_=(γ ○ *S*′_Tx_)′: received current signal/pulse, where ○ represents the convolution operator. *R*_xy_(τ): correlation result from UWB device.

The question arising in this context is the following: what is the minimum change of the tumour’s dielectric properties induced by the contrast agent during wash-in/wash-out in order to be detectable by UWB radar. To address this question we propose an analytical model of the transmit/receive channel including the signal processing part of the UWB unit [20]. This allows one to predict the effects of the permittivity changes on the UWB time courses. In the following, we will apply this model on the specific biomedical problem formed by an intracranial tumour located directly underneath the cortical bone (Figure 11, anatomical slice of the human head does not indicate the tumour position).

### 6.1. The Model

The set-up used to probe the human head with a UWB device is depicted in Figure 11. The antennas are co-polarized and normal incidence of the EM-wave is assumed. The UWB signal, a pulse or a pseudo-noise sequence of up to 10 GHz bandwidth, is transmitted utilizing appropriate pulse-radiating antennas. The reflected signal is detected by Rx and calculating the correlation *R*_xy_(τ), between received signal *S*_Rx_ and transmitted signal pulse *S*_Tx_ is usually the first step in further signal-processing. The head is treated as a multilayered dielectric structure with a characteristic reflection coefficient Γ(ω), modelled from nine planar isotropic dielectric layers. The arrangement as well as individual thicknesses of these layers approximate a trans-cranial slice from the Visual Human data set [19] (Figure 11), neglecting diffraction and refraction.

**Figure 12.**
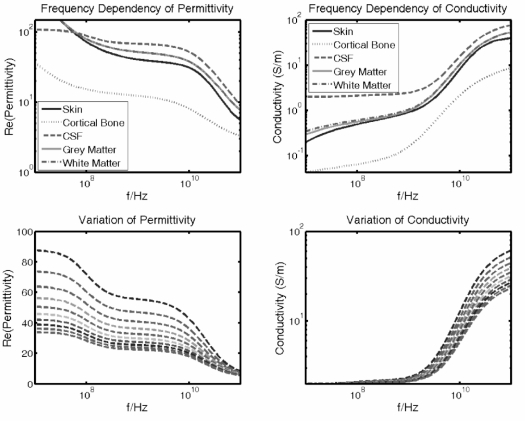
*Two upper images*: Frequency dependency of permittivity (real part: ε′) and conductivity σ of the different tissues forming the human head model. *Two lower images Lower:* Permittivity and conductivity variation of layer 3 representing the tumour (nine steps decreasing from ε′_1_ = 55 down to ε′_9_ = 22 at 1 GHz).

The spectral response of a dielectric medium is appropriately described in terms of multiple Cole-Cole dispersion which, with an appropriate choice of parameters for each constituent, can be used to predict the dielectric behaviour over the desired frequency range [21]. In Figure 12 the frequency dependence of the different biological tissues’ permittivity ε′ and conductivity σ is depicted in the frequency range of 100 MHz to 100 GHz.

The lower part of Figure 12 depicts the change of ε′ and σ in layer 3 starting from ε′_1_ = 55 down to ε′_9_ = 22 at 1 GHz. The change can be thought of as originated by the maximum concentration of different contrast agents or by the enrichment dynamic within the tumour (in the latter case a contrast agent with low ε′ is assumed). For such a layered arrangement the reflection response Γ(ω*,* ε) can be recursively calculated using an iterative formulation given in Reference [22]. In the frequency domain, the E-field ratios at the Rx and Tx antennas can be calculated and thus the received signal *S*_Rx_ can be derived [20].

**Figure 13.**
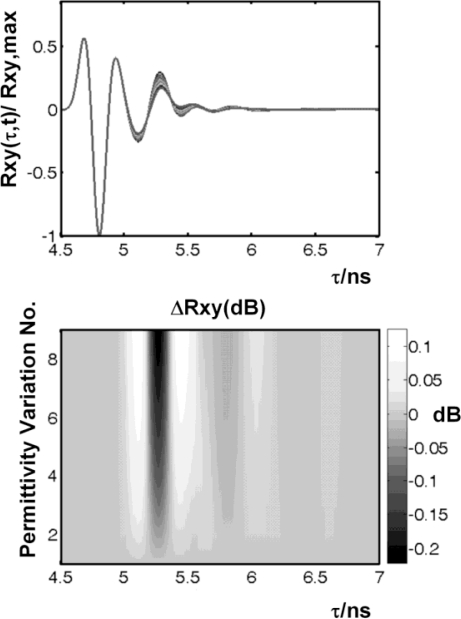
*Top*: correlation results *R*_xy_(τ*,*ε*_i_*)/*R*_xy_,_max_ gained from variation of dielectric properties (nine different frequency courses, steps). *Bottom*: overlapped variation Δ*R*_xy_(τ*,*ε*_i_*), i = 1,…,9.

**Figure 14.**
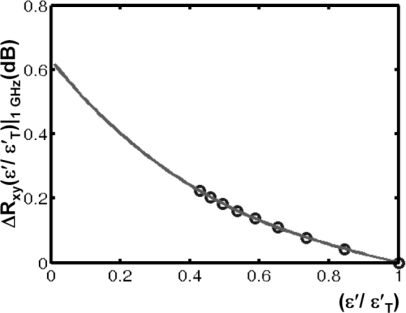
Variation of Δ*R*_xy_ *= R*_xy_(τ,ε*_i_*)/*R*_xy_(τ,ε*_1_*) in dB. *Circles:* stepwise variation of permittivity according to Figure 12. *Curve:* ΔR*_xy_* fit of model values for 0.01< ε′/ε′_T_ < 1, stepsize:0.01.

In the following the simulation results based on the variation of Γ(ω*,* ε_i_) are presented. The correlation result *R*_xy_(τ*,*ε*_i_*) and the variation Δ*R*_xy_(τ*,*ε*_i_*) = *R*_xy_(τ*,*ε*_i_*)/*R*_xy_(τ*,*ε*_1_*) after a certain propagation time are depicted in Figure 13. A a permittivity ratio ε′/ε′_T_(1 GHz) ∼ 0.67 (see Figure 14) is required in the tumour tissue to achieve a variation in *R*_xy_ exceeding 0.1 dB at 1 GHz. This corresponds to a 30% decrease in the tumour’s permittivity due to the contrast agent.

A fit of the model values for 0.01< ε′/ε′_T_<1 is given by ( step-size: 0.01, see Figure 14):

(3) $${\Delta R_{xy,\text{max}}\left(\frac{{ɛ}^{\prime }}{{ɛ}_{T}^{\prime }}\right)|}_{1GHz}=-0.3371\cdot {\left(\frac{{ɛ}^{\prime }}{{ɛ}_{T}^{\prime }}\right)}^{3}+1.0428\cdot {\left(\frac{{ɛ}^{\prime }}{{ɛ}_{T}^{\prime }}\right)}^{2}-1.335\cdot \left(\frac{{ɛ}^{\prime }}{{ɛ}_{T}^{\prime }}\right)+0.6292$$

We conclude that the detection of intracranial tumours close to the skull using non-contact UWB and contrast-enhancing agents is indeed feasible [36]. It surely requires an application method which supports the local enhancement of the contrast agent while circumvent the transport through, e.g., the liver. Due to the high change in dielectric properties which is needed, non-toxicity must be guaranteed. Finally, for this model the same simplifying assumptions are valid, which lead to the same points raised at the end of Section 3 to be necessarily considered if real measurements are going to be performed.

## 7. Simulation of the Electromagnetic Field Distribution

**Figure 15.**
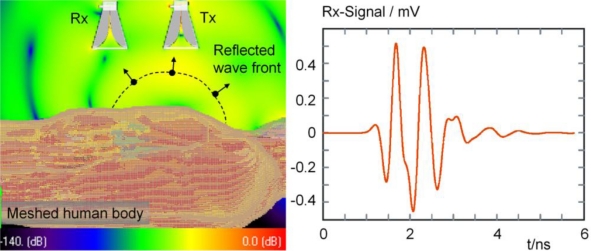
*Left*: Simulated wave propagation with reflected wave front. *Right*: Simulated output signal of the receiving antenna after excitation with a Gaussian pulse.

Beside the analytical approach we are interested in the temporal evolution of the electromagnetic field inside and outside the human thorax. In this regard we have investigated complex arrangements mimicking the illumination of a realistic human torso model incorporating the geometry of the antennas by finite-difference time-domain method (FDTD) simulations. A model of an adult male from the virtual family [37] was utilized for that purpose (see Figure 15, left). From such a simulated human body model the reflected signals can be gained (Figure 15, right), which are accordingly shaped to the antennas transfer characteristic and the subject’s reflection coefficient as analytically derived in Equations 1 and 2. An example comprising 16 million voxels is presented in Figure 15 on the left.

The human body is meshed by voxels of 2 mm × 2 mm × 2 mm. To keep the details of the antennas, described in [17], the mesh is locally refined to an edge length of 0.5 mm. A Gaussian pulse of 250 ps width, generated by a current source, was applied for the excitation of the transmission antenna in this example. By FDTD simulation, we can investigate, e.g., the dependence of the illumination and detection angles of the transmission and receiving antenna on the quality of the received signal, *i.e.*, the correlation result (Equation 2). In this regard, an estimate of the optimized antenna placement can be found. Furthermore, by varying organs’ boundaries by changing their thickness or/and placement of one or more tissue layers, different selected functional states, e.g., the end-systolic and end-diastolic phase of the myocardium, can be investigated, which consequently determines a characteristic change of the reflected signals in the antenna output.

**Figure 16.**
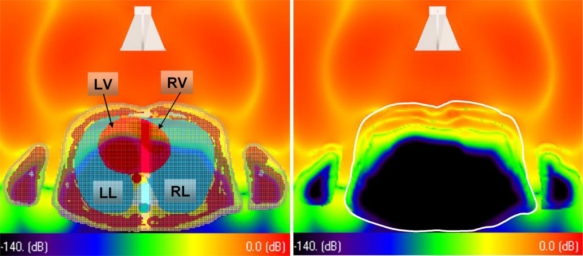
Field distribution in an axial cross section extra- and intra-corporal. *Left*: with tissue mesh. *Right*: without the mesh, showing the wave propagation intra-corporal. The thorax’s contour is highlighted by the white line.

An example of the complex wave propagation inside the human torso is shown in Figure 16. Due to the elevated permittivity ε inside the body the propagation velocity is slowed down according to *c* = *c*_0_/√ε. Hence, the bending of the extra- and intra-corporal wave fronts. The transmitted spherical wave fronts seem to be refracted towards the centre of the thorax, which is beneficial for the illumination of the myocardial section lying deeper inside the thorax.

By these simulations, we will achieve an in depth understanding of the complex electromagnetic field distribution and the dependencies of the resulting output signal of the receiving antenna. Due to the fact, that the deeper the interface of interest the worse the resolution of the reflection signal due to signal damping and dispersion [38]. The results of these simulations are therefore beneficial to increase the accuracy of reconstructed physiological signatures from deep sources by finding the optimized antenna positioning regarding the better penetrability of selected body areas. This of course requires the adaptation of the model to the actual thorax geometry of the patient which can be provided from MRI scans.

## 8. Summary

We have shown that ultra-wideband sensors, in combination with an appropriate signal processing technique can be beneficial in many fields of biomedical research. We focused especially on the conservation of the enhanced resolution capabilities of high- and ultrahigh filed magnetic resonance imaging and the enhancement of cardiac MRI by the proposed technique. The latter is based upon our research which has proven that with this ultra-wideband technique the mechanical equivalent to the electrocardiogram and furthermore the mechanical deformation of different functional units of the myocardium can be rendered and can be used for time gating of an MR system. These results encouraged the application of UWB radar in the field of MR safety, *i.e*., for the evaluation of the influence of very high static magnetic fields (≥3 T) on myocardial mechanics.

Optimization of data acquisition by the use of UWB antenna arrays to localize the motion in a focused area, will improve the result. Possible additional applications beside the proposed ones could be infarction detection, for ischemic tissue results in a modified contraction pattern, potentially accessible by UWB radar. Another patho-physiological event, detectable by our technique, could be electromechanical dissociation, which refers to any heart rhythm observed in the electrocardiogram that should be producing a pulse, but is not. A further application could be the identification of the mechanical equivalent to the QT interval, for recent guidelines drafts of the International Conference on Harmonisation of Technical Requirements for Registration of Pharmaceuticals for Human Use (ICH) underline the necessity to test non-antiarrhythmic drugs for their potential to prolong the QT or the corrected QT (QTc) interval [39,40]. The implementation of these guidelines requires a large amount of ECG or MCG (magnetocardiography) [41] measurements on animals and humans in preclinical and clinical phases of the drug development process. We propose the use of UWB radar as a complementary method with particular advantages in high-throughput studies, where signal quality and reliability are key factors.

Beside the experimental approaches we gained meaningful insights into the individual biomedical challenge by the implementation of a sound analytical approach seeking to model the different scenarios. From this approach we obtained encouraging results suggesting that intracranial oscillations also should be detectible by our method. Based on this model, we tried to answer the question whether the vascularisation dynamic of a contrast agent perfused tumour can be rendered by UWB radar. Our analytical scenario of an intracranial tumour perfused by a hypothetic contrast agent exhibiting varying dielectric properties proves the feasibility of this approach.

Further steps are the investigation of the electromagnetic field propagation inside and outside the human body by FDTD simulations. In this way a sound understanding of the forward problem and the signals received by the antennas as well as their intrinsic influence can be gained. On this basis the extraction and the interpretation of meaningful physiological signatures by adapted signal processing techniques will be improved.

This work was supported by *German Research Foundation* (DFG) priority program SPP1202 UKoLoS, (*ultra*MEDIS). The authors are grateful to M. A. Hein, J. Sachs, R. Stephan, U. Schwarz and M. Helbig from *Technische Universität Ilmenau*. We would also like to thank our medical partners I. Hilger, C. Geyer, G. Rimkus and W. A. Kaiser from the Institute for Diagnostic and Interventional Radiology (*FSU Jena*).
